# Development of a self-replicating plasmid system for *Mycoplasma hyopneumoniae*

**DOI:** 10.1186/1297-9716-44-63

**Published:** 2013-07-29

**Authors:** Gareth A Maglennon, Beth S Cook, Dominic Matthews, Alannah S Deeney, Janine T Bossé, Paul R Langford, Duncan J Maskell, Alexander W Tucker, Brendan W Wren, Andrew N Rycroft

**Affiliations:** 1 Department of Pathology and Pathogen Biology, The Royal Veterinary College, Hawkshead Lane, North Mymms, Hatfield AL9 7TA, United Kingdom; 2 Section of Paediatrics, Imperial College London, St Mary’s Campus, London W2 1PG, United Kingdom; 3 Department of Veterinary Medicine, University of Cambridge, Madingley Road, Cambridge CB3 0ES, United Kingdom; 4 Department of Pathogen Molecular Biology, London School of Hygiene & Tropical Medicine, Keppel Street, London WC1E 7HT, United Kingdom

## Abstract

*Mycoplasma hyopneumoniae* is a prevalent swine respiratory pathogen that is a major cause of economic loss to pig producers. Control is achieved by a combination of antimicrobials, vaccination and management practices, but current vaccines offer only partial control and there is a need for improved preventative strategies. A major barrier to advances in understanding the pathogenesis of *M. hyopneumoniae* and in developing new vaccines is the lack of tools to genetically manipulate the organism. We describe the development and optimisation of the first successful plasmid-based system for the genetic manipulation of *M. hyopneumoniae*. Our artificial plasmids contain the origin of replication (*oriC*) of *M. hyopneumoniae* along with *tetM*, conferring resistance to tetracycline. With these plasmids, we have successfully transformed *M. hyopneumoniae* strain 232 by electroporation, generating tetracycline resistant organisms. The persistence of extrachromosomal plasmid and maintenance of plasmid DNA over serial passages shows that these artificial plasmids are capable of self-replication in *M. hyopneumoniae*. In addition to demonstrating the amenability of *M. hyopneumoniae* to genetic manipulation and in optimising the conditions necessary for successful transformation, we have used this system to determine the minimum functional *oriC* of *M. hyopneumoniae*. In doing so, we have developed a plasmid with a small *oriC* that is stably maintained over multiple passages that may be useful in generating targeted gene disruptions. In conclusion, we have generated a set of plasmids that will be valuable in studies of *M. hyopneumoniae* pathogenesis and provide a major step forward in the study of this important swine pathogen.

## Introduction

Bacteria in the genus *Mycoplasma* are characterised by their lack of a cell wall, small genome size and utility in synthetic biology. They are responsible for a number of important diseases in humans and other animals. Among the most important species is *Mycoplasma hyopneumoniae* that is the cause of enzootic pneumonia, a chronic swine respiratory disease of global economic importance to pig producers [1]. Partial control of *M. hyopneumoniae* is achieved by a combination of management practices, antimicrobials and immunisation with commercially available vaccines [1]. Although current vaccines improve performance parameters and reduce the severity of lung lesions, there is a need for more effective vaccines, particularly in response to concerns regarding the current over-use of antimicrobials in production animals. Progress in understanding the molecular basis of *M. hyopneumoniae* pathogenesis is essential for improved intervention measures such as vaccine development, but this has been severely hampered by the lack of tools to genetically manipulate the organism [2]. Such tools may prove valuable in identifying and studying determinants of virulence in the mycoplasma genome that are central to the ability of the organism to cause disease. Some advances have been made with other mycoplasmas using transposon mutagenesis to generate random insertional mutants that can be screened for the expression of virulence factors using in vivo or in vitro model systems [3-9]. Such techniques have not been reported to date for *M. hyopneumoniae*. Additionally, transposon mutagenesis techniques produce random insertions into the mycoplasma genome, thus it can be difficult to isolate mutants with disruptions in particular genes of interest. A few mycoplasmas have also been transformed with artificial plasmids that are capable of self-replication [10-14]. These plasmids contain the origin of replication (*oriC*) of the mycoplasma along with an antimicrobial resistance gene cassette to facilitate genetic selection. Following transformation, these plasmids are initially replicated extrachromosomally, but can undergo integration into the host cell chromosome by recombination, owing to homology between the plasmid *oriC* and the *oriC* of the mycoplasma chromosome. In addition to demonstrating the amenability of mycoplasmas to genetic manipulation, in some cases *oriC*-based plasmids have been useful in promoting the generation of mutations in target genes by homologous recombination [11,12]. Plasmid stability can be increased by reducing the size of the plasmid *oriC* to the minimum that is functional, therefore reducing the likelihood of incorporation of the plasmid by homologous recombination into the host chromosome [11,12]. DNA sequences homologous to genes of interest in the mycoplasma genome could then be incorporated into the plasmid, and used to promote homologous recombination at specific sites in the mycoplasma chromosome.

In this study, a simple *oriC*-based plasmid system was developed as a method of determining the susceptibility of *M. hyopneumoniae* to transformation and optimising the conditions necessary for transformation. Requiring only the *M. hyopneumoniae* origin of replication and a suitable antimicrobial selection marker, we anticipated that such simple plasmids would be more likely to achieve successful transformation than, for example, a transposon mutagenesis system that also requires successful transposition into the host cell chromosome. The ability to transform *M. hyopneumoniae* has been long anticipated. Here we describe the generation of an *oriC*-based system for *M. hyopneumoniae* that facilitates the genetic transformation of this important pathogen.

## Materials and methods

### Bacterial strains and culture

*Mycoplasma hyopneumoniae* strain 232 [2] was grown in Friis medium as described by Kobisch and Friis [15]. Liquid cultures were grown at 37 °C in a static incubator in airtight culture tubes. For growth on solid medium, a final concentration of 0.8% w/v purified agar was added to Friis medium and plates were incubated at 37 °C with 5% CO_2_. Colonies were visualised by light microscopy at ×35 magnification using a stereoscopic microscope. For the selection of transformed *M. hyopneumoniae*, tetracycline hydrochloride (Sigma-Aldrich Ltd, Gillingham, UK) or puromycin dihydrochloride (Sigma-Aldrich Ltd, Gillingham, UK) were added to a final concentration of 0.2–2.0 μg/mL and 5 μg/mL respectively. For molecular cloning, *Escherichia coli* strain DH5α (Invitrogen Ltd, Paisley, UK) was grown in Luria-Bertani medium containing 50 μg/mL ampicillin at 37 °C with shaking at 220 rpm. For the selection of organisms expressing the tetracycline resistance gene, *tetM*, 5 μg/mL tetracycline hydrochloride was also added to solid medium.

### Transformation of *M. hyopneumoniae*

*Mycoplasma hyopneumoniae* strain 232 was grown in liquid culture until mid/late logarithmic phase (~10^8^ CFU/mL), as determined by an acid colour change in the phenol red pH indicator. Cells were collected by centrifugation at 9000 × *g* at 4 °C for 10 min and were washed three times in an equivalent volume of electroporation buffer (272 mM sucrose, 8 mM HEPES, pH 7.4). After the final wash cells were re-suspended in electroporation buffer such that 100 μL cells was equivalent to approximately 2 mL of mycoplasma culture. Mycoplasmas were incubated on ice with plasmid DNA for 30 min and then transferred to a pre-chilled 0.2 cm electroporation cuvette (Bio-Rad Ltd, Hemel Hempstead, UK). Electroporation was performed using a Gene Pulser system (Bio-Rad Ltd, Hemel Hempstead, UK). Standard electroporation conditions were 2.5 kV, 100 Ω and 25 μF. Immediately after electroporation, 900 μL chilled Friis medium were added. After incubation for a further 15 min on ice, cells were transferred to a microcentrifuge tube and incubated for a further 3 h at 37 °C. Cultures were then plated onto solid Friis medium containing 0.2–2.0 μg/mL tetracycline and were incubated at 37 °C in 5% CO_2_ for 10–14 days.

### Plasmid construction

**Figure 1:**
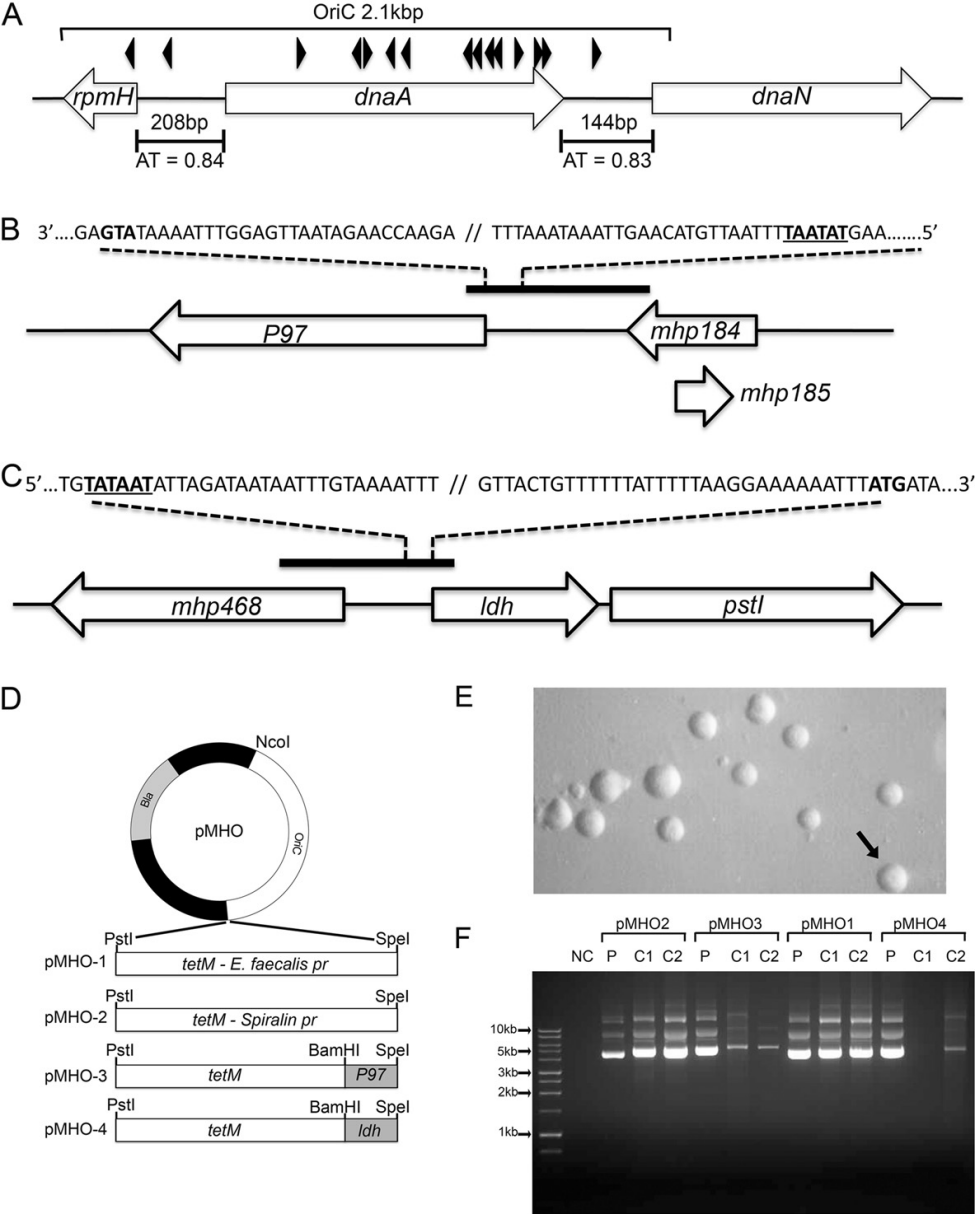
***oriC *plasmid construction.** A 2.1 kbp *oriC* region of *M. hyopneumoniae* strain 232 was predicted based on the location of putative DnaA boxes (black arrowheads), the location of *dnaA* and the presence of short AT-rich regions of 144 bp and 208 bp with an AT content of 0.83 and 0.84 respectively **(A)**. The *P97* gene **(B)** and *ldh* gene **(C)** promoter sequences were predicted based on proximity to the ATG start codon (shown in bold) and putative TATA box location (underlined). The *oriC* sequence was cloned into the *Nco*I and *Spe*I restriction sites of pGEM-T to generate plasmid pMHO. The *tetM* gene with the *E. faecalis*, spiralin gene, *P97* and *ldh* promoter sequence was cloned into the *Pst*I and *Spe*I sites to produce plasmids pMHO-1, pMHO-2, pMHO-3 and pMHO-4 respectively **(D)**. *M. hyopneumoniae* strain 232 was transformed with each plasmid and transformants *(pMHO-2* in **E**) grown in Friis medium with tetracycline selection. After 3 passages, plasmid DNA was extracted from two individual clones (C1 and C2) and analysed by agarose gel electrophoresis along with plasmid (P) DNA control **(F)**.

**Figure 2:**
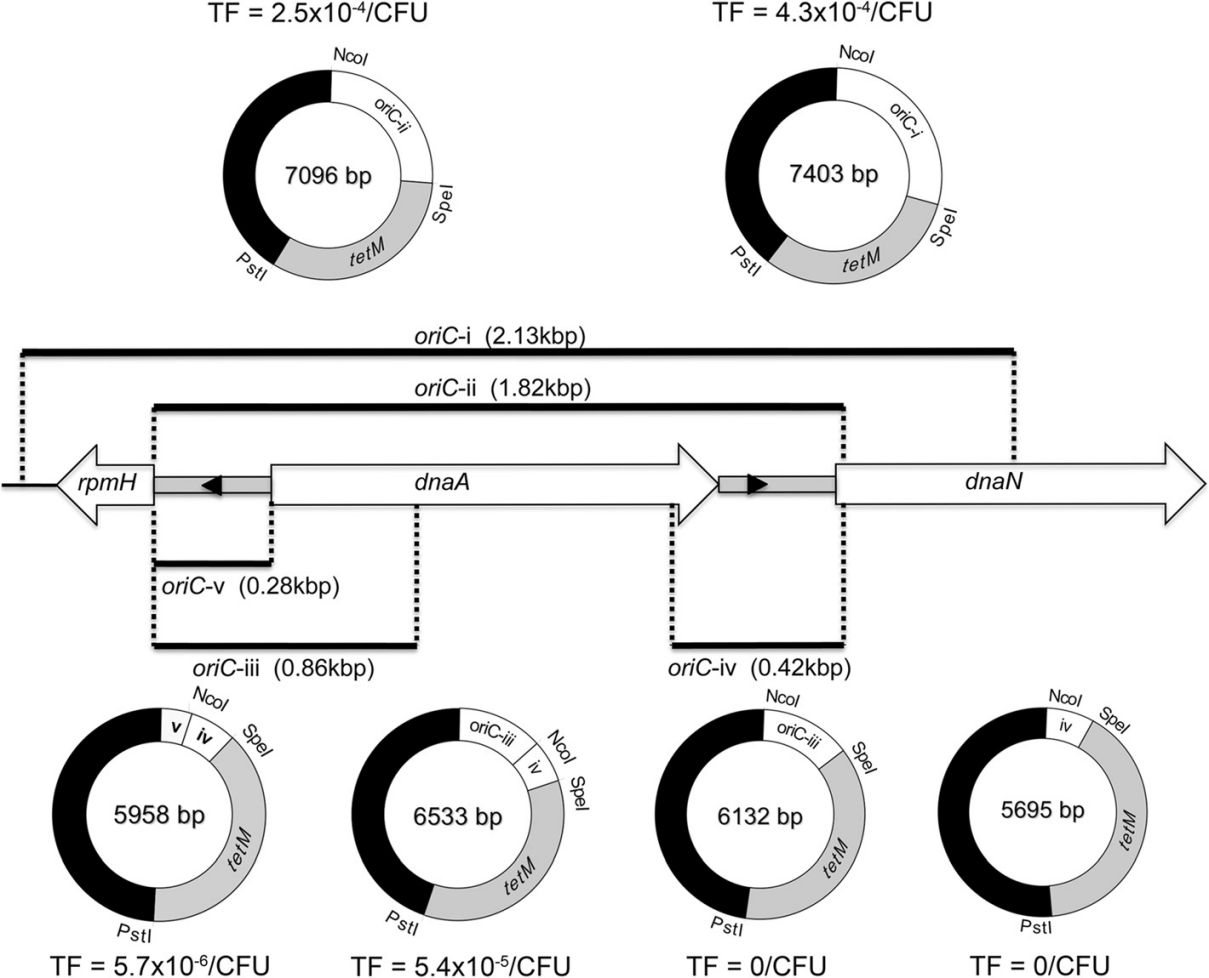
**Minimum *oriC *determination.** The predicted *oriC* region of *M. hyopneumoniae* strain 232 is shown with the location of two putative DnaA boxes (black arrowheads) lying within AT-rich intergenic regions (grey boxes). The *oriC* region from plasmid pMHO (oriC-i) was reduced in a series of steps to produce five further plasmids (pOriC-ii, pOriC-iii, pOriC-iv, pOriC-iii/iv and pOriC-iv/v). *M. hyopneumoniae* strain 232 was transformed in triplicate with each plasmid and the mean transformation efficiencies (TF) are shown, expressed as the number of transformants per colony forming unit of *mycoplasma* transformed.

Sequences of oligonucleotide primers used in the construction of plasmids are shown in Table 1. Polymerase chain reaction (PCR) was performed using a Phusion High-Fidelity DNA Polymerase kit (NEB Ltd, Hitchin, UK) according to the manufacturers instructions. PCR-amplified DNA products were cloned into plasmids at restriction sites added to oligonucleotides as shown in Table 1 and in plasmids as shown in Figures 1 and 2. The *oriC* of *M. hyopneumoniae* strain 232 was predicted based on nucleotide sequence similarity with other *Mycoplasma spp.* for which a functional *oriC* has been verified [11,12,14]. Typically, the *oriC* encompasses short AT-rich regions flanking *dnaA* and containing DnaA boxes. A search of the *M. hyopneumoniae* strain 232 genome [GenBank: AE017332] revealed two DnaA boxes (each with two bp mismatches with the *Escherichia coli* consensus sequence 5′-TTATCCACA-3′) upstream and downstream of the *dnaA* gene within AT-rich regions (Figure 1A). Plasmid pMHO (Figure 1D) was generated by cloning a PCR-amplified 2.1 kbp sequence encompassing *dnaA* and the AT-rich regions flanking each end of the gene into the pGEM-T cloning vector (Promega Ltd, Southampton, UK). The *tetM* gene and promoter from *Enterococcus faecalis* were amplified from plasmid pIVT-1 [5] and cloned into pMHO to produce pMHO-1. To generate pMHO-2, the *tetM* gene from the plasmid pSRT2 [11] was amplified by PCR and cloned into pMHO. The *tetM* gene of pMHO-2 contains the promoter sequence of the spiralin gene of *Spiroplasma citri*. To generate plasmids pMHO-3 and pMHO-4, the *tetM* gene minus the spiralin gene promoter sequence was amplified from pSRT2 and cloned into pMHO. The predicted promoter regions of the P97 ciliary adhesin gene (619 bp) and the lactate dehydrogenase (ldh) gene (421 bp) were amplified by PCR from *M. hyopneumoniae* strain 232 and placed upstream of the *tetM* gene start codon to produce plasmids pMHO-3 and pMHO-4 respectively. The *oriC* regions of *M. hyorhinis* [GenBank: CP002170.1] [16], *M. pulmonis* [GenBank: NC_002771.1] [17], *M. conjunctivae* [GenBank: NC_012806.1] [18] and *M. ovipneumoniae* [GenBank: AFHO00000000.1] [19] were determined using the same criteria as for *M. hyopneumoniae* (location of *dnaA* gene, AT-rich sequences and DnaA boxes) and by reference to published studies [11]. *oriC* regions were amplified by PCR and used to replace the *M. hyopneumoniae* strain 232 *oriC* region of pMHO-2, to produce the plasmids pMhyor, pMpulm, pMconj and pMovip. Plasmids containing shortened regions of the *M. hyopneumoniae* strain 232 *oriC* were constructed as shown in Figure 2. Shortened *oriC* regions were amplified by PCR from *M. hyopneumoniae* strain 232 genomic DNA and used to replace the *oriC* region of pMHO-2 to generate plasmids pOriC-ii, pOriC-iii, pOriC-iv, pOriC-iii/iv and pOriC-iv/v. Some of our *M. hyopneumoniae* field isolates are more resistant to tetracycline than others. Therefore, as an alternative selection marker to *tetM* we chose the puromycin N-acetyltransferase (*pac*) gene conferring resistance to puromycin. pMHO-puro was constructed by inserting the *pac* gene from pMini-Tn*4001*PsPuro [GenBank: FJ872396] [20] into the *Pst*I cut site of plasmid pMHO. This *pac* gene also contains the spiralin gene promoter of *S. citri*.

**Table 1:** Oligonucleotide sequences.

**PCR product**	**Size (bp)**	**Restriction site**	**Oligonucleotide sequence (5′ - 3′)**
*M. hyopneumoniae* strain 232 *oriC*- i	2130	*Nco*I	*F* - AGG*CCATGG*TTGTTAATTATTGCTTGAAATTC
		*Spe*I	*R* - AGT*ACTAGT*AAACTTTATAGGAAAGTTCG
*M. hyopneumoniae* strain 232 *oriC*- ii	1823	*Nco*I	*F* - GATG*CCATGG*TTGGTTGATAAGTTCGCTTC
		*Spe*I	*R* - GTAG*ACTAGT*CATTCTTTCAATTTGTCATTC
*M. hyopneumoniae* strain 232 *oriC*- iii	859	*Nco*I	*F* - GATG*CCATGG*TTGGTTGATAAGTTCGCTTC
		*Spe*I	*R* - GCTC*ACTAGT*GACAATCAGTTTAAAAATTCAC
*M. hyopneumoniae* strain 232 *oriC*- iii	859	*Aat*II	*F* - GCAG*GACGTC*TTGGTTGATAAGTTCGCTCC
		*Nco*I	*R* - GACG*CCATGG*GACAATCAGTTTAAAAATTCAC
*M. hyopneumoniae* strain 232 *oriC*- iv	422	*Nco*I	*F* - GCAT*CCATGG*ATATTGTGCATGCCCGCGGATAT
		*Spe*I	*R* - GTAG*ACTAGT*CATTCTTTCAATTTGTCATTC
*M. hyopneumoniae* strain 232 *oriC*- v	280	*Aat*II	*F* - GCAG*GACGTC*TTGGTTGATAAGTTCGCTCC
		*Nco*I	R - CGTA*CCATGG*CCTAATTTGCTGTGTTCTAA
*tetM* + spiralin promoter from plasmid pSRT2	2320	*Pst*I	*F* - TAA*CTGCAG*CAAAAGCTTGCATGCCTGCA
		*Spe*I	*R* - GTC*ACTAGT*CAGTTCAGATCTTTATATAAC
*tetM* from plasmid pIVT	2109	*Spe*I	*F* - ATCT*ACTAGT*CATGTGATTCTAAAGTATCC
		*Pst*I	*R* - GAT*CTGCAG*CTTATTTAACAAGAAACC
*tetM* (without promoter) from plasmid pSRT2	1963	*Bam*HI/*Pst*I	*F* - GAA*CTGCAGGATCC*ATGGAGGAAAATCACATGA
		*Sal*I	*R* - CCCGGTCGACTTATATAACAACTTAAATTAC
*M. hyopneumoniae* strain 232 *P97* promoter	619	*Bam*HI	*F* - GAATGGATCCCCAACAATTCCGGCAGTC
		*Spe*I	*R* - GCTA*ACTAGT*ACGGGGATTTAAAACAGAAAC
*M. hyopneumoniae* strain 232 *ldh* promoter	421	*Spe*I	*F* - GGCTA*ACTAGT*ATAGAATTTTGCAATTAAAG
		*Bam*HI	*R* - CTTA*GGATCC*ATTTATACTCGTATTTGTTAT
*M. hyorhinis oriC*	1479	*Nco*I	F - GACT*CCATGG*TCGGAATCGAGATTCCTAATT
		*Spe*I	R - GCCT*ACTAGT*CTACACCTTCGATTTCTCTAA
*M. pulmonis oriC*	1909	*Nco*I	F - ATTAG*CCATGG*CACTCTGGTCAGCGCTAGATC
		*Nhe*I	R - TCGC*GCTAGC*CTAACTTGAAAATAAGCTCC
*M. conjunctivae oriC*	2307	*Nco*I	F - GATA*CCATGG*TATGCCTTTGGTCTAGGATG
		*Xba*I	R - CGATG*TCTAGA*CCCTTGAACAAATATACCA
*M. ovipneumoniae oriC*	2102	*Nco*I	F – AGTCCATGGTCAATTATTGCTTGGAATTC
		*Spe*I	R - CGTACTAGTAAACTTTATAAGAAAGTTCGCT
*bla* gene (DIG-labelled probe)	860	N/A	F – CCAATGCTTAATCAGTGAGG
			R - GTATGAGTATTCAACATTTCCG
*tetM (DIG-labelled probe)*	406	N/A	F – GTGGACAAAGGTACAACGAG
			R - CGGTAAAGTTCGTCACACAC

Oligonucleotide sequences are shown along with the size of the PCR product generated and the template DNA used. Restriction enzyme sites (where applicable) that were added to the 5′ end of the oligonucleotide are shown in italics. Forward (F) and reverse (R) oligonucleotides are shown.

### Southern hybridisation

Total DNA was extracted from 20 mL mycoplasma broth culture using a Qiagen DNA Blood & Tissue kit (Qiagen Ltd, Manchester, UK) and 1 μg was digested to completion with *Hin*dIII. DNA was separated by electrophoresis on 0.8% agarose and blotted onto Hybond-N + membrane (GE Healthcare Ltd, Little Chalfont, UK) using standard methods [21]. DNA was fixed to the membrane by exposure to UV light. DIG-labelled probes specific for the *bla* ampicillin resistance gene of pGEM-T or the *tetM* gene were generated from PCR-amplified DNA (see Table 1 for oligonucleotide sequences) using a DIG-High Prime DNA Labelling and Detection Starter Kit II (Roche Applied Science Ltd, Burgess Hill, UK). The same kit was used to perform pre-hybridisation and hybridisation in accordance with the manufacturers instructions. The membrane was autoradiographed at room temperature using CL-XPosure Film (Fisher Scientific Ltd, Loughborough, UK).

## Results

### Generation of *M. hyopneumoniae* transformants

In our initial attempts at transforming *M. hyopneumoniae*, we constructed a simple plasmid containing the predicted *oriC* region of *M. hyopneumoniae* strain 232 and *tetM* conferring resistance to tetracycline. A large 2.1 kbp region of the *M. hyopneumoniae* strain 232 genome encompassing the entire *dnaA* gene and upstream and downstream AT-rich regions was selected. Homologous DNA sequences of other mycoplasmas demonstrating a similar gene arrangement have shown success in functioning as an *oriC* in artificial self-replicating plasmids [9,11]. The *tetM* gene has been used as a selection marker for mycoplasmas [10,12] and we confirmed the sensitivity of *M. hyopneumoniae* strain 232 to tetracycline. Following the methods of Hannan [22], the minimum inhibitory concentration (MIC) of tetracycline was 0.03 μg/mL in liquid medium and 0.015 μg/mL in solid medium. Following the transformation of *M. hyopneumoniae* strain 232 with 10 μg of pMHO-1, cells were incubated for 3 h at 37 °C and then plated onto Friis agar containing 2 μg/mL tetracycline. Triplicate repeats were performed and after 14 days incubation, no growth was observed for pMHO-1 or “no DNA” controls. We considered that the *tetM* promoter sequence may be inactive in *M. hyopneumoniae* and/or that the tetracycline concentration may be too high to be overcome by transformed organisms. The *tetM* sequence under control of its own promoter, as derived from *Enterococcus faecalis*, is expressed at levels sufficient to confer tetracycline resistance in several mycoplasmas [12,23-25]. However, other mycoplasmas may require an alternative promoter sequence, such as the *spiralin* gene promoter of *Spiroplasma citri* that has be shown to be active in several mycoplasma species [11,13,14]. In addition to constructing pMHO-2 utilising *tetM* and the spiralin gene promoter sequence, we produced plasmids pMHO-3 and pMHO-4 containing *tetM* with the putative promoter regions of the *M. hyopneumoniae* strain 232 P97 ciliary adhesin gene [26] and lactate dehydrogenase (*ldh*) gene respectively. In addition to altering the promoter sequences controlling *tetM* expression, we sought to determine whether or not a lower concentration of tetracycline would enhance the growth of transformants. For other mycoplasmas, transformed cells are typically selected using concentrations of between 2 and 4 μg/mL tetracycline [12,23,25]. Having determined that a concentration of 0.015 μg/mL could inhibit growth of *M. hyopneumoniae* 232 on Friis agar, we lowered the concentration of tetracycline to 0.2 μg/mL. *M. hyopneumoniae* strain 232 was transformed in triplicate with plasmids pMHO-1, pMHO-2, pMHO-3 and pMHO-4 and plated onto Friis agar containing 0.2 μg/mL tetracycline. After incubation for 14 days tetracycline resistant colonies were present in bacteria transformed with all four *oriC* plasmids but were completely absent in the “no DNA” control, demonstrating activity of the four promoter sequences. Morphologically, colonies were identical to untransformed *M. hyopneumoniae* colonies, but colony growth lagged by approximately 3 days (Figure 1E). The highest number of transformants was generated using pMHO-2 (2.5 × 10^-4^/CFU), with fewer for pMHO-3 (5.8 × 10^-5^/CFU) and pMHO-1 (1.7 × 10^-5^/CFU), and least with pMHO-4 (8.3 × 10^-6^/CFU). Individual colonies were picked from agar and grown in Friis medium containing 0.5 μg/mL tetracycline, after which they were sub-cultured every 2–3 days in 1 μg/mL tetracycline. After three serial passages, plasmid DNA was extracted and separated by electrophoresis. Plasmid DNA was readily identified for each transformant whereas no plasmid DNA was seen with untransformed *M. hyopneumoniae* 232 (Figure 1F). However, less plasmid DNA was detected for pMHO-3 and pMHO-4 transformants. Given that both of these plasmids contain promoter sequences 400–600 bp in size that are identical to *M. hyopneumoniae* genomic DNA, we suspect that plasmid integration had occurred by homologous recombination.

### Optimisation of transformation conditions

Initially, we selected electroporation conditions of 2.5 kV, 100 Ω and 25 μF and used a cuvette with a 2 mm gap as described by Hedreyda et al. [7]. We varied the electroporation voltage from a maximum possible of 2.5 kV down to 0 kV in 500 V increments and used pMHO-2 to transform *M. hyopneumoniae* 232. Electroporation voltage was found to have a profound effect on transformation frequency; decreasing voltage to 2 kV resulted in a 10-fold drop in transformation frequency from 5.4 × 10^-6^ CFU/mL to 4.2 × 10^-7^ CFU/mL, and below 2 kV no transformants were obtained (Table 2). Similarly, decreasing plasmid DNA was associated with a drop in transformation frequency; a drop from 10 μg down to 1 μg resulted in a 10-fold drop in transformants from 5.6 × 10^-3^ CFU/mL to 2.5 × 10^-4^ CFU/mL (Table 3). Attempts to improve transformation frequency further by increasing the DNA concentration above 10 μg typically resulted in arcing.

**Table 2:** Effect of electroporation voltage on transformation frequency.

**Electroporation voltage**	**Average transformation frequency (transformants/CFU) [SE]**
2.5 kV	5.4 × 10^-6^ [1.2 × 10^-6^]
2.0 kV	4.2 × 10^-7^ [3.9 × 10^-7^]
1.5 kV	0
1.0 kV	0
0 kV	0

The effect of altering the electroporation voltage on transformation frequency was examined. *M. hyopneumoniae* strain 232 was transformed with pMHO-2 and the average transformation frequencies (with standard error) of three separate transformations are shown.

**Table 3:** Effect of plasmid DNA concentration on transformation frequency.

**DNA quantity**	**Average transformation frequency (transformants/CFU) [SE]**
10 μg	5.6 × 10^-3^ [1.1 × 10^-3^]
5 μg	5.0 × 10^-3^ [1.4 × 10^-3^]
2 μg	3.3 × 10^-4^ [9.6 × 10^-5^]
1 μg	2.5 × 10^-4^ [3.7 × 10^-5^]
0 μg	0

The effect of plasmid DNA concentration of transformation frequency was examined by transforming *M. hyopneumoniae* strain 232 with different quantities of pMHO-2 DNA. Average transformation frequencies (with standard error) of three transformations are shown.

### Host species specificity of *oriC* regions

Suspecting that the large 2.1 kbp *oriC* in our plasmids would favour integration by homologous recombination, we determined whether the predicted *oriC* regions of four phylogenetically closely related *Mycoplasma* species would function in *M. hyopneumoniae*. We hypothesised that the *oriC* regions of one or more of these mycoplasmas may be sufficiently similar to that of *M. hyopneumoniae* to be recognised as an *oriC*, yet different enough that the incidence of homologous recombination would be reduced. The genomes of *M. hyorhinis*[16], *M. pulmonis*[17], *M. conjunctivae*[18] and *M. ovipneumoniae*[19] exhibited a similar arrangement of genes in the *oriC* region to *M. hyopneumoniae*, with AT-rich regions and DnaA boxes in the intergenic regions flanking *dnaA*. *M. hyopneumoniae* strain 232 cells were electroporated in triplicate with plasmids pMHO-2, pMhyor, pMpulm, pMconj and pMovip in two independent experiments and cultured on Friis agar containing 0.2 μg/mL tetracycline. As expected, many individual tetracycline resistant colonies were obtained with the pMHO-2 plasmid within 10 days but not for “no DNA” controls. However, after 14 days, no colonies were visible in any culture transformed with plasmids containing the *oriC* regions of the other *Mycoplasma* species.

### Determination of the minimum functional *oriC* of *M. hyopneumoniae*

As an alternative strategy to reduce the incidence of homologous recombination, we attempted to reduce the size of *oriC* used in our plasmids. To define the minimum functional *oriC* in *M. hyopneumoniae*, initially the *oriC* of pMHO-2 (*oriC*-i) was reduced by 300 bp by removing excess DNA sequence lying upstream and downstream of the AT-rich intergenic regions (pOriC-ii). With both AT-rich regions and the *dnaA* gene still present, as expected, there was no reduction in transformation efficiency (Figure 2). Next, we determined whether each of the two AT-rich intergenic regions flanking the *dnaA* gene would function as an *oriC* independently. Plasmids pOriC-iii and pOriC-iv were constructed containing the upstream and downstream intergenic regions respectively. pOriC-iii contained the AT-rich region upstream of *dnaA*, and also extended downstream into *dnaA* to include three further putative DnaA boxes. *M. hyopneumoniae* strain 232 was transformed in triplicate with each plasmid but failed to produce any transformants. Therefore, each AT-rich region was unable to function independently as an *oriC*. However, by placing both intergenic regions adjacent to each other in plasmid pOriC-iii/iv, transformants were produced in all three triplicate transformations. The transformation efficiency was approximately 10-fold lower than when the entire *dnaA* gene was present (Figure 2). Finally, we constructed plasmid pOriC-iv/v to determine whether or not the entire *dnaA* gene sequence could be removed leaving the AT-rich intergenic regions alone. Following electroporation of *M. hyopneumoniae* strain 232 with pOriC-iv/v, tetracycline resistant colonies were generated but with a transformation efficiency approximately 100-fold lower than that achieved when the *dnaA* gene sequence was included (Figure 2). We concluded that the minimum *oriC* necessary for functionality in self-replicating plasmids was approximately 700 bp long and included both AT-rich intergenic regions lying upstream and downstream of the *dnaA* gene, but that the *dnaA* gene sequence itself was not essential to this. To determine how robust the resistance to tetracycline of pOriC-iv/v transformants was, we determined the MIC of three individual colonies in triplicate. After initial growth in 1 μg/mL, the MIC for tetracycline of all three clones in Friis broth was 32 μg/mL compared to 0.03 μg/mL for untransformed *M. hyopneumoniae* strain 232.

### Stability of plasmid DNA

**Figure 3:**
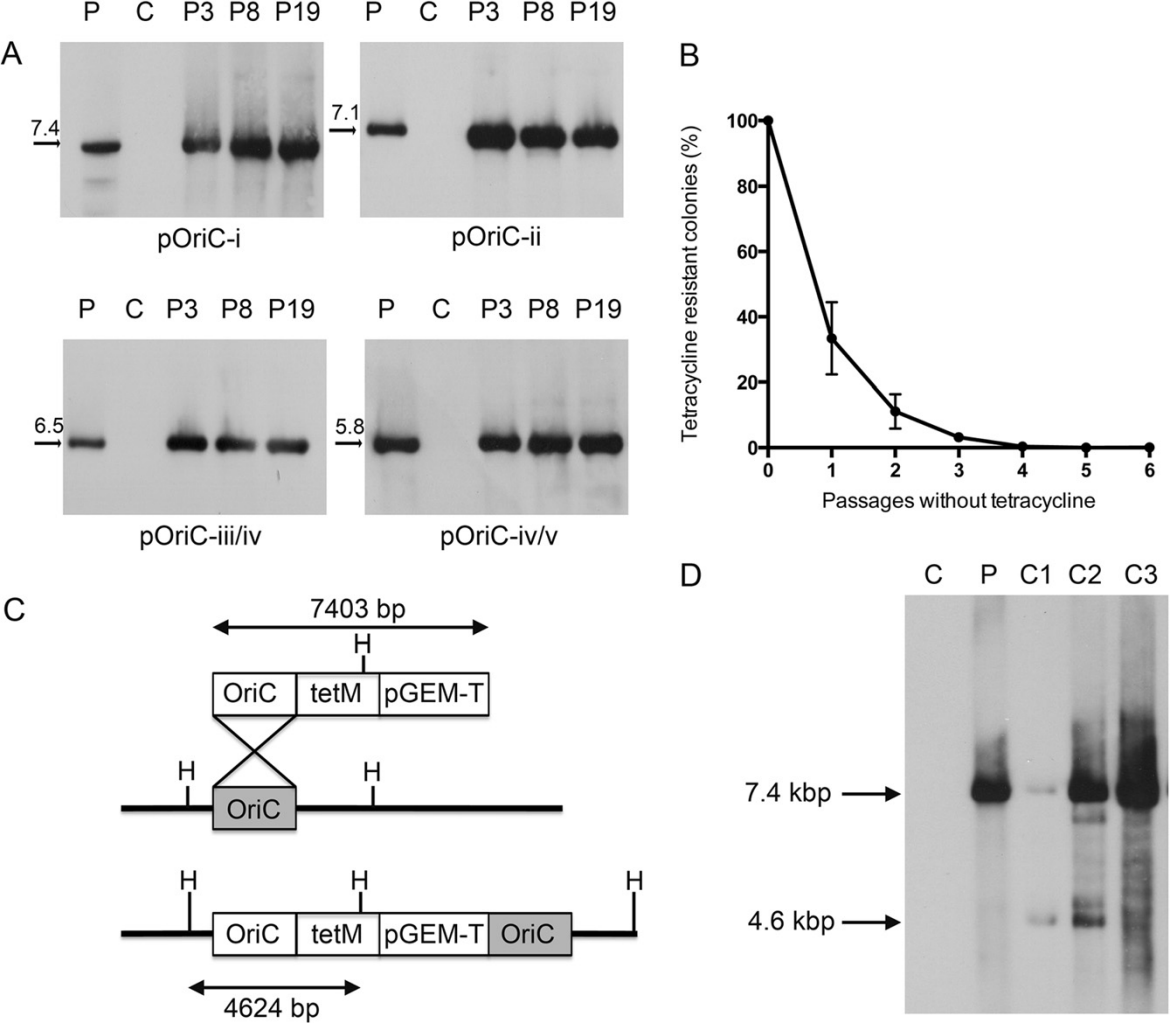
***oriC *plasmid stability.** Individual transformants generated with four plasmids containing various *oriC* regions were passaged up to 19 times in Friis medium containing tetracycline **(A)**. Genomic DNA was extracted from cultures and analysed by Southern hybridisation using a probe annealing to the *bla* ampicillin resistance gene. For each plasmid, there was no decline in extrachromosomal DNA over time, and plasmid DNA could still be readily detected after 19 passages. For plasmid pOriC-iv/v, three transformants were passaged up to six times without tetracycline before undergoing selection with tetracycline **(B)**. With each passage without selection, the number of tetracycline-resistant colonies reduced, demonstrating a loss of tetracycline-resistance. Possible integration events were predicted for plasmid pMHO-2 containing *oriC*-i **(C)**. Three further pMHO-2 transformants were passaged 19 times and total DNA extracts subjected to Southern hybridisation with a *tetM* DIG-labelled probe, along with plasmid DNA control (P) and untransformed *M. hyopneumoniae* strain 232 DNA **(C) (D)**. Two clones showed the presence of 7.4 kbp plasmid DNA and also a 4.6 kbp fragment consistent with plasmid integration by homologous recombination.

To determine the stability of our plasmids upon serial passage over time, individual transformants were picked into Friis medium containing 0.5 μg/mL tetracycline and passaged 19 times. At passages 3, 8 and 19, total DNA was extracted and Southern hybridisation performed with a DIG-labelled probed specific for the *bla* ampicillin resistance gene of pGEM-T. For each plasmid, we could readily detect extrachromosomal DNA at every passage, but not for the untransformed *M. hyopneumoniae* strain 232 control (Figure 3A). Additionally, we did not observe a reduction in plasmid DNA quantity that may be suggestive of plasmid integration into the *M. hyopneumoniae* genome by homologous recombination. We further determined the stability of the minimal *oriC* plasmid, pOriC-iv/v, over serial passages without selection. Three individual transformants were passaged five times in Friis medium with 0.5 μg/mL tetracycline followed by six passages without tetracycline. At each of the six passages without selection, cultures were plated (in triplicate) onto Friis agar with and without 0.2 μg/mL tetracycline. After 14 days growth, colony counts were performed. We observed a rapid reduction in tetracycline resistant colonies following serial passage in medium containing no tetracycline, showing that selection is necessary to maintain resistance, and suggesting that the transformants can readily be “cured” of plasmid DNA (Figure 3B). We anticipate that this stable plasmid may be useful in generating targeted gene disruptions of *M. hyopneumoniae*. We therefore examined three clones of pMHO-2 (containing *oriC*-i) transformed *M. hyopneumoniae* at passage 19. We predicted that integration of plasmid pMHO-2 into the *M. hyopneumoniae* strain 232 genome would generate a 4.6 kbp *Hin*dIII digestion fragment that would be detected by a DIG-labelled probe annealing to the *tetM* gene. By separating a larger amount (2.5 μg) of *Hin*dIII-digested total DNA and using a DIG-labelled *tetM* gene probe, we were able to visualise a 7.4 kbp band consistent with plasmid DNA in three transformants and a 4.6 kbp band consistent with integrated plasmid DNA in two transformants. This would suggest that recombinase systems active in *M. hyopneumoniae* are capable of allowing homologous recombination to occur, and therefore the minimum *oriC* plasmid may be useful in directing homologous recombination to target genes. Finally, we found that many of our UK field isolates exhibited greater resistance to tetracycline than *M. hyopneumoniae* 232, making it unachievable to select for transformed colonies on Friis agar containing 0.2 μg/mL tetracycline (data not shown). To circumvent this problem, we replaced the *tetM* gene of pMHO-2 with the puromycin N-acetyltransferase (*pac*) gene from pMiniTn*4001*PsPuro [20] to generate plasmid pMHOpuro. This *pac* gene also contains the spiralin gene promoter of *S. citri* and confers resistance to puromycin, an aminonucleoside that has no veterinary clinical use, and therefore will be generally applicable as a genetic marker for *M. hyopneumoniae*. Transformation of *M. hyopneumoniae* strain 232 with pMHOpuro resulted in a transformation frequency of 1.6 × 10^-4^/CFU compared to 4.6 × 10^-5^ for plasmid pMHO-2 (containing the *tetM* gene). Thus puromycin may be a suitable selection marker for *M. hyopneumoniae* isolates that are resistant to tetracycline.

## Discussion

The inability to genetically manipulate *M. hyopneumoniae* has been a major obstacle to progress in understanding the survival and pathogenesis of the organism. Using simple artificial self-replicating plasmids containing the *oriC* of *M. hyopneumoniae* and an antimicrobial resistance gene, we have shown for the first time that the organism is susceptible to transformation. We found that the transformation conditions optimised by Hedreyda et al. [7] and used widely in other *Mycoplasmas* are suitable for *M. hyopneumoniae*. However, other critical factors include titration of the tetracycline concentration to a level that can readily be overcome by the TetM resistance protein, the use of high voltage for electroporation and the use of a large excess of plasmid DNA. Additionally, we found that the choice of promoter affected the expression of the *tetM* gene, and as is the case with other mycoplasmas, the spiralin gene promoter of *Spiroplasma citri* is a suitable choice for *M. hyopneumoniae*. Our *oriC* system allowed us to assess the function of putative *M. hyopneumoniae* promoter sequences, and confirmed the identity of two promoter sequences that were active in controlling *tetM* expression. The *P97* promoter sequence was recently predicted and we have verified that this is functional in our plasmid system [26]. Using the criteria identified by Weber Sde et al. [26], we also predicted the location of the *ldh* promoter, and again demonstrated its activity in our plasmid system. These *M. hyopneumoniae*-specific promoters will be valuable in the generation of further tools for the genetic manipulation of the organism. Using our system, we verified that the origin of replication of *M. hyopneumoniae* lies in the region of *dnaA* and includes two short AT-rich regions. It has been suspected that the *oriC* of *M. hyopneumoniae* lies in this region, but unusually within the AT-rich regions there are only two DnaA boxes that resemble the *E. coli* consensus sequence, whereas for other mycoplasma species, there are typically multiple DnaA boxes [2]. Additional DnaA boxes are present within the *dnaA* gene, but these are not required for a functional *oriC*. We hypothesised that the *oriC* regions of mycoplasmas showing a high degree of nucleotide sequence similarity to *M. hyopneumoniae* would function in *M. hyopneumoniae*, and enable stable plasmid maintenance without integration by homologous recombination. However, it appears that *oriC*s are very specific to their host species and none of the *oriC* regions from four closely related *Mycoplasma* species functioned in *M. hyopneumoniae*.

The minimum *M. hyopneumoniae* sequence that functioned in an *oriC* plasmid (pOriC-iv/v) was approximately 700 bp in length and included both AT-rich regions. This plasmid was maintained extrachromosomally over at least 19 passages with selection pressure, equivalent to over 8 weeks post-transformation. However, when selection pressure was removed, tetracycline resistance was rapidly lost within 5 passages. Having demonstrated that plasmids carrying sequences with a high degree of homology to regions of the *M. hyopneumoniae* genome can undergo integration by homologous recombination, it is likely that this plasmid will be useful in generating targeted disruptions in genes of interest. By incorporating sequences encoding mycoplasma genes of interest, it is likely that we will be able to direct homologous recombination and cause disruption of these genes, as has been shown with other mycoplasmas [11,12]. In addition to *tetM*, we demonstrated that *pac* conferring resistance to puromycin can be expressed in *M. hyopneumoniae* strain 232 from our *oriC* plasmids when driven by the spiralin gene promoter sequence of *S. citri*. While *M. hyopneumoniae* strain 232 is exquisitely sensitive to tetracycline, as demonstrated in this study, many of our recent field isolates of *M. hyopneumoniae* are relatively resistant (data not shown). However, these strains are susceptible to puromycin, therefore *pac* may be a more suitable selection marker for generating transformants in other *M. hyopneumoniae* isolates.

In summary, we have shown that *M. hyopneumoniae* is indeed susceptible to genetic manipulation by transformation with self-replicating plasmids. In doing so, we have developed a system that will undoubtedly be useful in genetic studies of *M. hyopneumoniae* and probably other mycoplasma species. Additionally, we anticipate that our findings will be a key step towards the development of a transposon-mediated system to generate random mutants of *M. hyopneumoniae*, which will take us a step closer to unraveling the pathogenesis of this poorly understood pathogen.

## Competing interests

The authors declare that they have no competing interests.

## Author’s contributions

Experiments were conceived by GAM, ANR & BSC. Experiments were performed by GAM, ASD & DM. BSC, JTB, ANR, PL, BWW, AWT and DJM assisted with experimental design. The manuscript was written by GAM & ANR and was approved by all authors.

## Supplementary Material

Additional file 1 **BRaDP1T consortium.** List of researchers collaborating in the BRaDP1T consortium. Click here for file
