# Supplementary material for: Development of a self-replicating plasmid system for Mycoplasma hyopneumoniae
Source: Vet Res. 2013 Jul 29;44(1):63. doi: 10.1186/1297-9716-44-63 (PMC3765554; doi:10.1186/1297-9716-44-63)
Supplement: Additional file 1 — BRaDP1T consortium. List of researchers collaborating in the BRaDP1T consortium. [file 1297-9716-44-63-S1.doc]

**Royal Veterinary College:**

Andrew N Rycroft

Gareth A Maglennon

Dominic Matthews

**Imperial College London:**

Paul R Langford

Janine T Bossé

Yanwen Li

**Cambridge University Veterinary School:**

Duncan J Maskell

Sarah Peters

Lucy Weinert

AW (Dan) Tucker

Tracy Wang

Shi L Luan

Roy Chaudhuri

**London School of Hygiene & Tropical Medicine:**

Brendan W Wren

Jon Cuccui

Vanessa Terra
